# Effects of estradiol on biological age measured using the glycan age index

**DOI:** 10.18632/aging.104060

**Published:** 2020-10-13

**Authors:** Julija Jurić, Wendy M. Kohrt, Domagoj Kifer, Kathleen M Gavin, Marija Pezer, Peter A. Nigrovic, Gordan Lauc

**Affiliations:** 1 Genos Glycoscience Research Laboratory, Zagreb, Croatia; 2 Division of Geriatric Medicine, School of Medicine, University of Colorado Anschutz Medical Campus, Aurora, CO 80045, USA; 3 Eastern Colorado VA Geriatric Research, Education and Clinical Center, Aurora, CO 80045, USA; 4 Department of Biochemistry and Molecular Biology, Faculty of Pharmacy and Biochemistry, University of Zagreb, Zagreb, Croatia; 5 Division of Rheumatology, Inflammation, and Immunity, Brigham and Women´s Hospital, Boston, MA 02115, USA; 6 Division of Immunology, Boston Children´s Hospital, Boston, MA 02115, USA

**Keywords:** biological age, glycan age, estrogen, aging biomarkers, glycosylation

## Abstract

Glycan age is a recently developed biomarker based on glycans attached to immunoglobulin G (IgG). In large population cohorts, glycan age associates well with lifestyle and disease-risk biomarkers, while some studies suggested that glycan changes precede development of several age-associated diseases. In this study we evaluated effects of estrogen on the glycan age. Gonadal hormones were suppressed in 36 healthy young women by gonadotropin releasing hormone agonist therapy for 6 months. In 15 of them estradiol was supplemented, while 21 received placebo resulting in very low estrogen levels during intervention. IgG was isolated from plasma samples before intervention, after 6 months of intervention and after subsequent 4-month recovery. Deprivation of gonadal hormones resulted in median increase of glycan age for 9.1 years (IQR 6.8 – 11.5 years, p = 3.73×10^-8^), which was completely prevented by transdermal estradiol therapy (change in glycan age = -0.23 years, IQR (-2.20 – 2.98). After the recovery period glycan age returned to baseline values in both groups. These results suggest that IgG glycans and consequently also the glycan age are under strong influence of gonadal hormones and that estradiol therapy can prevent the increase of glycan age that occurs in the perimenopausal period.

## INTRODUCTION

The existence of inter-individual differences in the pace of biological aging is an intriguing concept that tries to explain why some people stay healthy until very late chronological age, while other people age faster and have a shorter life expectancy. A number of biomarkers aimed at an objective estimation of biological age have been developed in the past several years, one of them being the glycan age, which is based on analyzing glycans attached to immunoglobulin G (IgG) [1]. A key feature of a good biomarker of biological age is that the difference between chronological and biological age should correlate with known biomarkers of an unhealthy lifestyle and that increased biological age should predict future disease development. Glycans attached to IgG change significantly with age [1] and have been suggested as a promising biomarker of biological age [2]. Furthermore, since glycosylation affects interactions between IgG and different Fcγ receptors and other ligands, changes in glycosylation have direct effects on the function of the immune system [3], with multiple functional implications.

The decrease in IgG galactosylation was first reported over 35 years ago in patients with rheumatoid arthritis and osteoarthritis [4]. This was subsequently confirmed in multiple studies, which also reported that it not only associated with disease activity and progression, but also predicted response to therapy and preceded the development of the disease for up to several years [5]. Decreased IgG galactosylation was reported to associate with many other autoimmune diseases including juvenile onset rheumatoid arthritis, systemic lupus erythematosus, Crohn’s disease, ulcerative colitis, Sjögren’s syndrome, neonatal lupus, coeliac disease and myasthenia gravis [6]. Most of the studies related to IgG glycosylation alterations during aging reported that early adulthood IgG glycosylation is characterized by the highest abundance of digalactosylated and the lowest amount of agalactosylated structures, and with aging a decrease in galactosylation and an increase in agalactosylation can be seen [6]. IgG glycans have been shown to be a reliable biomarker of aging that explained up to 64% of variation in chronological age [1, 7]. However, IgG glycans are not only biomarkers but also functional effectors that participate in the process of aging. According to the inflammaging concept, the age-related gradual decrease in IgG galactosylation level due to chronic low-grade sterile inflammation in the elderly exacerbates inflammation, creating a feedback loop in which the agalactosylated IgG species represent both a biomarker of aging and a contributor to its pathogenesis [8, 9].

Large population studies [1, 10] and our recent study of an intervention cohort suggest that estrogens regulates IgG glycosylation [11], which may explain why IgG glycome in premenopausal females reflects apparent lower biological age. Unfortunately, the published estrogen intervention study was based on the analysis of glycans released from all proteins in the plasma proteome, thus it was not possible to reliably differentiate IgG glycans from glycans released from other proteins. This prevented the calculation of glycan age from the available data since glycan age is based on IgG glycans. Aiming to evaluate the effects of ovarian sex hormone suppression followed by estradiol supplementation on biological age measured by the glycan age we reanalysed samples from the same intervention study using state of the art glycoprofiling technology [12].

## RESULTS

**Figure 1:**
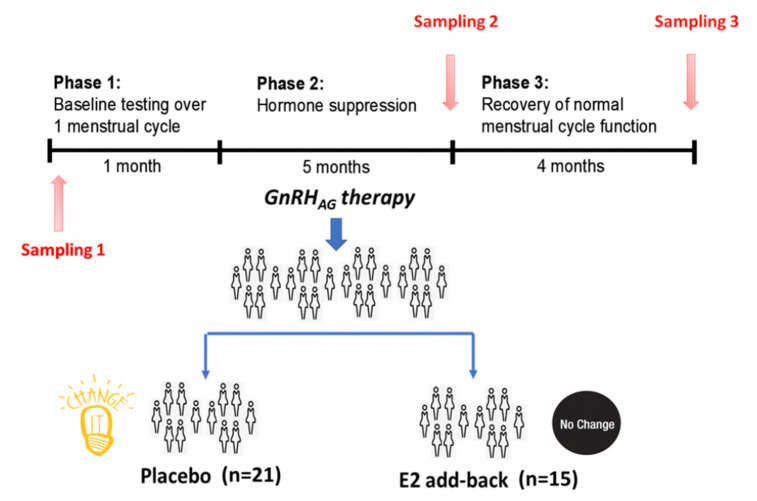
**Design of the gonadal hormone suppression intervention study.**

IgG glycosylation was analyzed in 36 healthy premenopausal women that were treated on an investigational basis with the gonadotropin-releasing hormone (GnRH) analogue leuprolide to lower gonadal steroids to postmenopausal levels and then randomized to placebo or transdermal estradiol (Figure 1) [13]. Plasma samples were collected at baseline (T1), after five months of hormonal suppression by monthly leuprolide injections plus either estradiol or placebo patches (T2), and four months after the end of intervention when natural hormonal cycling was restored (T3). The concentration of hormones at the baseline and differences from the baseline after intervention and after recovery timepoint are presented in Table 1.

**Table 1:** The concentration of hormones at baseline and differences from baseline after the intervention and at recovery.

**Variable**	**Intervention**	**Concentration At baseline median (IQR)**	**Difference in concentration relative to baseline**			
			**Intervention median (IQR)**	**p_I_**	**Recovery median (IQR)**	**p_R_**
**estradiol** ***pg/mL***	Placebo	54.0 (44.5 - 79.2)	-31.5 (-53.5 to -19.8)	0.001	-7.5 (-45.2 to 25.5)	0.989
	Estradiol	57 (46 - 78)	-15.0 (-35.0 to 29.5)		-1 (-32 to 30)	
**estrone** ***pg/mL***	Placebo	52 (37 - 67)	-19 (-32 to -11)	0.001	-1 (-16 to 13)	0.989
	Estradiol	55.0 (39.0 – 68.0)	3.0 (-10.5 to 20.0)		0.0 (-10.5 to 23.0)	
**FSH** ***mIU/mL***	Placebo	5.95 (4.40 - 8.02)	-1.10 (-3.35 to -0.05)	0.001	0.00 (-1.15 to 1.40)	0.471
	Estradiol	6.60 (4.95 - 9.15)	-5.10 (-7.25 to -3.05)		-1.85 (-3.03 to 0.45)	
**LH** ***mIU/mL***	Placebo	4.60 (3.55 - 5.10)	-4.00 (-4.88 to -2.88)	0.768	-0.700 (-1.450 to 0.725)	0.815
	Estradiol	4.90 (3.00 - 6.55)	-4.70 (-6.05 to -2.70)		-0.70 (-3.35 to 0.35)	
**progesterone** ***ng/mL***	Placebo	0.4 (0.3 - 0.7)	-0.1 (-0.4 to 0.0)	0.989	0.0 (-0.2 to 0.2)	0.760
	Estradiol	0.4 (0.3 - 0.6)	-0.1 (-0.2 to 0.0)		0.1 (-0.2 to 0.6)	
**SHBG** ***nmol/L***	Placebo	52 (30 - 63)	-8 (-16 to -2)	0.094	2 (-6 to 5)	0.760
	Estradiol	35.0 (29.5 - 54.0)	-2.0 (-6.5 to 8.5)		-1.0 (-5.0 to 1.5)	
**testosterone** ***ng/dL***	Placebo	28.0 (17.0 - 32.2)	-4.0 (-10.8 to 0.0)	0.760	0.0 (-2.0 to 6.9)	0.760
	Estradiol	29.0 (23.5 - 35.5)	-4.0 (-8.5 to 1.0)		0.0 (-3.0 to 7.0)	

p values describe statistical significance of difference between estradiol and placebo group after intervention (p_I_) and recovery (p_R_). p values smaller than 0.05 in bold.

IQR – limits of the interquartile range (1^st^ quartile - 3^rd^ quartile).

FSH - Follicle-stimulating hormone; LH - Luteinizing hormone; SHBG - sex hormone-binding globulin.

**Figure 2:**
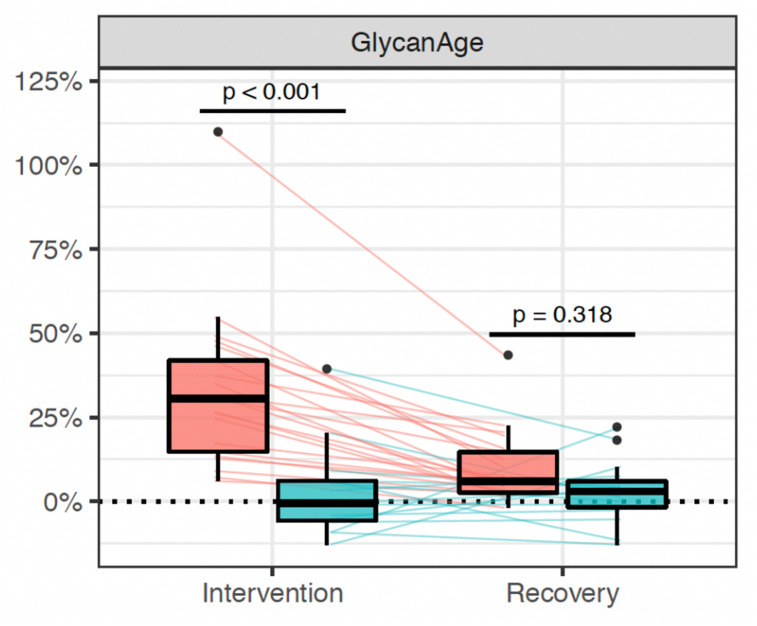
**Distribution of changes in glycan age in 36 women undergoing gonadal hormone suppression for 6 months.** Statistically significant increase in glycan age was observed in the placebo group (n=25, red rectangle), while supplementation with estradiol prevented this change (n = 15, blue rectangle).

**Figure 3:**
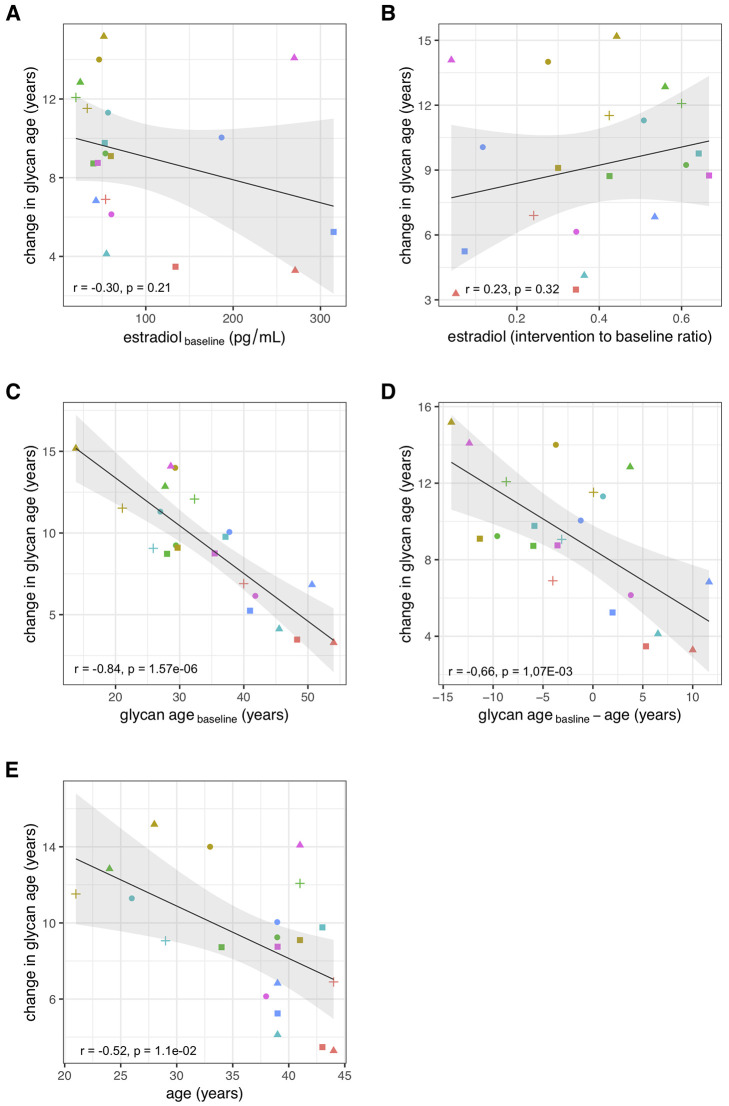
Correlations between the change in glycan age caused by gonadal hormone suppression and baseline estradiol concentration (**A**), change in estradiol concentration (**B**), glycan age (**C**), change in glycan age (**D**) and age (**E**).

Suppression of ovarian sex hormones production resulted in a median increase of glycan age by 9.1 years, which was completely abolished by estradiol therapy (Figure 2, Table 2). Both the extent of change in hormone levels (Table 1) and the extent of change in glycan age varied significantly, thus we wondered whether the estradiol baseline levels or the extent of changes in estradiol levels correlated with the extent of change in glycan age. The analysis did not reveal any statistically significant correlation between these two parameters (Figures 3A and 3B). Then we checked whether the change in glycan age correlated with baseline chronological age, baseline glycan age or the difference between chronological and glycan ages. Intuitively one would expect a larger increase in glycan age in chronologically younger women and indeed we observes an inverse correlation between the extent of change induced by suppression of gonadal hormones and age (r = -0.54, p = 1.1**×**10^-2^, Figure 3E). Interestingly, much stronger correlation was observed for the initial glycan age (r = -0.84, p = 1.57**×**10^-6^, Figure 3C) and the difference between glycan age and the chronological age (r = -0,66, p = 1,07**×**10^-3^, Figure 3D) suggesting that low glycan age is strongly dependent on gonadal hormones.

**Table 2:** Chronological age (years) at the baseline, and differences in glycan age relative to the baseline after the intervention and after recovery timepoint.

**Intervention**	**Age (years) At baseline Median (IQR)**	**Difference in glycan age (years) Relative to baseline. Sampling after:**			
		**Intervention Median (IQR)**	**P_i_**	**Recovery Median (IQR)**	**P_r_**
**PLACEBO (N = 21)**	39.0(33.0 – 41.0)	9.10(6.83 - 11.52)	3.73×10-8	2.31(1.19 - 4.34)	0.318
**ESTRADIOL (N = 15)**	38.0(29.5 – 43.5)	-0.23(-2.20 - 2.98)		1.31(-0.81 - 2.88)	

p values describe statistical significance of difference between estradiol and placebo group after intervention (p_I_) and recovery (p_R_).

## DISCUSSION

One of the key requirements for an aging biomarker is that it is responsive to interventions that beneficially affect the biology of aging, but convincing evidence of this is still missing for any aging biomarker [14]. In this study, we showed that the removal of gonadal hormones resulted in a rapid increase of glycan age, which was completely prevented by estradiol treatment, a therapy proven to be of benefit to some perimenopausal women [15]. Glycan age is a measure of biological age that is based on the analysis of IgG glycosylation [16]. Glycans attached to IgG are functionally important because they regulate inflammation at multiple levels [17, 18] and are considered to be one of the important drivers of inflammaging [9]. IgG glycosylation was reported to correlate with numerous unhealthy states and conditions including serum levels of glucose, insulin, hemoglobin A1c, triglycerides, total cholesterol, low-density lipoprotein, high-density lipoprotein, fibrinogen, d-dimer, uric acid, creatinine, alanine aminotransferase, aspartate aminotransferase and C reactive protein, as well as body mass index and waist circumference, systolic and diastolic blood pressure, smoking, hypertension, kidney function, diabetes and cardiovascular disease risk score [1, 19, 28, 20–27]. In addition, average heritability of the IgG glycome is estimated to be 55% [29, 30], which means the remaining variability is a result of environmental factors and different (patho)physiological variables related to age and lifestyle. However, the regulation and mechanisms underlying the age-related changes in IgG glycosylation remain mostly undiscovered, primarily due to the lack of research focusing on this question. Based on the results of many observational and molecular studies IgG glycans are proposed to play a role as both a biomarker and a functional contributor to the aging process, as well as to some age-related diseases. Here arises probably the most exciting aspect of the relationship between aging and IgG glycosylation: the potential of IgG glycans to distinguish between healthy and unhealthy aging, and to monitor the effect of introduced life-style changes on biological age.

Large studies of adult human populations indicated that IgG glycans without galactose and sialic acid that are the main component of the increased glycan age increase with the onset of menopause [1], while in girls they decrease with the onset of puberty [10, 31]. This indicated that estrogens may be relevant, but since many things change during puberty and menopause, the change in glycan age could not have been directly attributed to the change in estrogen concentration. In our study, suppression of gonadal hormones in premenopausal women resulted in a considerable (median 9.1 glycan age years) increase in glycan age that was statistically highly significant. Moreover, the change was observed in all study participants that received placebo treatment, and treatment with estradiol was sufficient to completely prevent the increase in biological age.

The extent of changes in both hormone levels (Table 1) and glycan age (Figure 2) varied considerably between individual participants. Aiming to determine what contributed to the extent of change within each individual, we evaluated the associations of changes in glycan age levels with basal hormone levels, changes in hormone levels, basal glycan age level and the difference between glycan age and chronological age. We did not find a significant correlation between the change in glycan age and the baseline serum estradiol concentration or the change in serum estradiol concentration after the intervention, indicating importance of some other unknown potential confounders or mediators. However, both basal glycan age and the difference between chronological and glycan age were strongly inversely correlated with the change in glycan age. This suggests that, despite being evidently important, estradiol is only one of the factors that define the glycan age of an individual.

Despite extensive research, progress in the development of biomarkers that could reliably quantify inter-individual differences in aging is still limited [32]. One of the important elements that is still missing is the ability to change the biomarker with lifestyle changes or pharmacological interventions. Recently a modest improvement in epigenetic age was reported in a small group of individuals undertaking quite radical pharmacological intervention [33] and glycan age was shown to slightly improve by exercise [34]. However, all these changes were modest compared to the effects of the suppression of gonadal hormones, which more than doubled glycan age in some of the participants. Treatment with estradiol was sufficient to completely abolish this effect. It is intriguing to speculate that hormone treatment could also prevent the increase of glycan age that occurs around menopause, but this still needs to be investigated. Furthermore, since IgG glycosylation is a functionally relevant modification that regulates the immune system, this discovery opens the option to look for downstream pathways that may be a more specific target for therapy than broadly acting estrogens.

## MATERIALS AND METHODS

### Institutional approval

This study was conducted at the University of Colorado Anschutz Medical Campus (CU-AMC). Procedures followed were in accordance with the ethical standards of and approved by the Colorado Multiple Institutional Review Board (COMIRB) and the Scientific Advisory and Review Committee at the University of Colorado Anschutz Medical Campus (CU-AMC). The study was registered on ClinicalTrials.gov (NCT00687739) on May 28, 2008.

### Participants and screening procedures

Participants were healthy eumenorrheic premenopausal women (n = 36). In accordance with the Declaration of Helsinki, volunteers provided written informed consent to participate, with the knowledge that the risks of the study included menopause-like effects (e.g., weight gain, bone loss, menopausal symptoms). Main inclusion criteria were age (25 to 49 y) and normal menstrual cycle function (no missed cycles in previous year, cycle length 28±5 d and confirmation of ovulatory status (ClearPlan Easy, Unipath Diagnostics, Waltham, MA)). Exclusion criteria were pregnancy or lactation, use of hormonal contraception, oral glucocorticoids, or diabetes medications, smoking, or body mass index (BMI) >39 kg/m^2^. Volunteers underwent screening procedures, as described previously [13].

### Experimental design and study procedures

The parent trial was a randomized, double-blinded, placebo-controlled trial to determine the effects of estradiol (E2) deficiency on body composition and energy expenditure, bone mineral density, components of energy expenditure and physical activity in premenopausal women [13, 35]. In short, all participants underwent suppression of ovarian sex hormones with gonadotropin releasing hormone agonist therapy (GnRH_AG_, leuprolide acetate 3.75 mg, Lupron; TAP Pharmaceutical Products, Inc; Lake Forest, IL) in the form of monthly intramuscular injections. A single injection of leuprolide acetate produces an initial stimulation (for 1 to 3 wk) followed by a prolonged suppression of pituitary gonadotropins FSH and LH, while repeated monthly dosing suppresses ovarian hormone secretion [36]. The absence of pregnancy was confirmed by a urine pregnancy test before each dosing. After screening procedures were completed, eligible volunteers underwent baseline testing during the early folicular phase (days 2 to 6 after onset of menses) of the menstrual cycle. At the beginning of the next menstrual cycle participants began with 5-month GnRH_AG_ therapy to chronically suppress ovarian function. Participants were randomized to receive either transdermal E2 0.075 mg/d (Bayer HealthCare Pharmaceuticals, Berkeley, CA) or placebo patches (GnRHAG+E2, n=15; GnRH_AG_+PL, n=21). In order to reduce the risk of endometrial hyperplasia, but in the same time minimizing the exposure to progesterone, women randomized to E2 received medroxyprogesterone acetate (5mg/d, as a pill) for 12 days every other month (end of month 2 and 4, and after completion of follow-up testing). During these monthly visits, participants were under supervision of the research nurse practitioner. Participants were asked to report changes in use of medications or health (e.g., doctor visits, hospitalizations), as well as any study-related problems/concerns over the past 4 weeks. The E_2_ regimen was expected to maintain serum E2 concentrations in the mid-to-late follicular phase range (100 to 150 pg/mL).

### Sample collection

Blood samples for sex hormones and glycans were collected in three timepoints: during baseline testing (T1), during week 20 of the hormonal intervention (T2), and at the spontaneous recovery of the normal menstrual cycle function (T3). A single sample (~5 mL) was obtained in the morning (~8 AM), after an overnight fast (at least 10 hours). Baseline samples were obtained immediately before the first GnRH_AG_ injection. Serum was separated from each collected sample upon blood withdrawal and stored at -80°C until analysis.

### Sex hormones

Collected sera were analyzed for numerous sex hormones. Estrone (E1), estradiol (E2) and progesterone (P) were determined by radioimmunoassay (RIA, Diagnostic Systems Lab, Webster, TX). Total testosterone (T) was analyzed by chemiluminescence immunoassay (Beckman Coulter, Inc. Fullerton, CA) and sex hormone-binding globulin (SHBG) by immunoradiometric assay (Diagnostic Systems Laboratory).

### N-glycosylation of immunoglobulin G

The whole procedure was performed according to already published protocol [29]. In short, IgG was isolated from sera samples by affinity chromatography using 96-well Protein G plate (BIA Separations, Slovenia). The isolated IgG was denaturated with the addition of SDS (Invitrogen, USA) and incubation at 65°C. The excess of SDS was neutralized by the addition of Igepal-CA630 (Sigma-Aldrich, USA) and N-glycans were released with the addition of PNGase F (Promega, USA) in PBS buffer followed by overnight incubation at 37°C. The released glycans were fluorescently labelled with 2-AB (Merck, Germany). Free label and reducing agent were removed from the samples by using hydrophilic interaction liquid chromatography solid phase extraction (HILIC-SPE). IgG N-glycans were eluted with ultrapure water and stored at -20°C until use. Fluorescently labelled N-glycans were separated using HILIC on an Acquity UPLC H Class Instrument (Waters, USA) that consists of sample manager, quaternary solvent manager and fluorescence (FLR) detector. The instrument was under the control of Empower 3 software, build 3471 (Waters, USA). Labelled N-glycans were separated on an amide ACQUITY UPLC® Glycan BEH chromatography column (Waters, USA), 100 x 2.1 mm i.d., 1.7 μm BEH particles, with 100 mM ammonium formate, pH 4.4, as solvent A and 100% ACN as solvent B. Samples were kept at 10°C before injection, and separation was performed at 60°C. The separation method used a linear gradient of 25-38% solvent A at a flow rate of 0.40 mL/min in a 27 min analytical run. Fluorescently labelled N-glycans were detected by FLR detector with excitation and emission wavelengths set at 250 and 428 nm. Data processing included an automatic integration algorithm that was manually corrected to maintain the same intervals of integration for all the samples. IgG N-glycan samples were all separated into 24 chromatographic peaks. The relative amount of glycans in each chromatographic peak was expressed as the percentage of the total integrated area (% Area).

### Statistical analysis

Area under chromatogram peaks was normalized to the total chromatogram area, then each glycan peak was logit transformed and batch corrected using ComBat method (R package ‘sva’) [37]. Data were back transformed, and derived glycan traits were calculated as a sum or ratio of selected directly measured glycan peaks based on particular glycosylation features (i.e. sialylation or fucosylation). GlycanAge was calculated according to Krištić et al. [1]: age model coefficients were trained using 1116 females (18 – 98 years old) from The Croatian National Biobank “10 001 Dalmatians” [38]. IgG N-glycome from the biobank was measured in the same laboratory and prepared in the same way as estrogen-study data. GlycanAge expressed in years was calculated using the following formula:

$$\begin{array}{l} \text{Glycan}\;\text{age}=56.08+776.01\times \text{GP}6-\\ 5376.83\;{\left(\text{GP}6\right)}^{2}-215.10\times \text{GP}14-30.70\times \text{GP}15 \end{array}$$

where GP<*n*> is *n*-th peak in chromatogram expressed as proportion of total chromatogram area.

Strength of the associations were estimated using Pearson’s correlation coefficient. All statistical analyses were performed in R programming software (version 3.6.3) [39].
